# Patients who restart antiretroviral medication after interruption remain at high risk of unfavorable outcomes in Ethiopia

**DOI:** 10.1186/s12913-017-2172-9

**Published:** 2017-04-04

**Authors:** Alula M. Teklu, Kesetebirhan D. Yirdaw

**Affiliations:** 1 St. Paul’s Hospital Millennium Medical College, Addis Ababa, Ethiopia; 2 MERQ Consultancy Services PLC, Addis Ababa, Ethiopia, Addis Ababa, Ethiopia; 3 University of South Africa, Addis Ababa, Ethiopia

**Keywords:** Treatment interruption, Antiretroviral medication, Lost, Tracking, Treatment Outcome, Ethiopia

## Abstract

**Background:**

Achieving optimal adherence to highly active antiretroviral therapy (HAART) is necessary to attain viral suppression and hence optimal clinical outcome. Interruptions in antiretroviral therapy medication often occur, but a substantial proportion restart treatment. Long-term care engagement practices and clinical outcomes have not been described among cohorts of individuals on HAART in Ethiopia.

**Methods:**

In this study we describe treatment interruption patterns over time among clients who interrupt and subsequently resume HAART, and those who are continuously engaged in treatment, and determine clinical factors associated with loss to engagement.

An observational, longitudinal, retrospective cohort design was engaged, using secondary treatment program data. We analyzed differences in treatment interruption among clients who were continuously active and those that interrupted and restarted treatment at months 6, 12, 18, and 24. Cox proportional hazards regression analysis was used to identify predictors of loss from treatment. We estimated time to first treatment interruption, time to restarting after interruption, and time to second interruption. Data from all clients registered to receive HAART in ten study health facilities, from 2005 to 2014, were used to study clinical and treatment outcomes up to 60 months or study end.

**Results:**

In this study, 39% (8,759/22,647) of clients interrupted treatment for more than 1 month at least at one point during follow-up. Of these, only 35% ever restarted treatment. At the end of follow-up, the hazard of unfavorable treatment outcome (dead, lost, stopped HAART) for clients who restarted treatment at months 6, 12, 18 and 24 was higher by a factor of 1.9, 2.4, 2.6 and 2.4, as compared to those who never discontinued treatment at those times.

**Conclusion:**

HAART treatment interruption was common in the study population. In those with a history of treatment interruption, long term clinical outcomes were found to be suboptimal. Targeted interventions are required to address follow-up challenges and prevent treatment interruption.

**Electronic supplementary material:**

The online version of this article (doi:10.1186/s12913-017-2172-9) contains supplementary material, which is available to authorized users.

## Background

Since the initiation of programs to provide free Highly Active Antiretroviral Therapy (HAART) in many low- and middle-income countries worldwide, the number of people living with HIV/AIDS who are receiving treatment has been increasing; reaching 17 million in 2015 [1]. Such programs have helped prevent mortality and new HIV infection among people irrespective of gender, age, race or economic status. The UNAIDS reports a decline in HIV/AIDS-associated mortality and in the rate of new HIV infection globally; an indication of both the success of HAART treatment programs and other methods to prevent disease transmission. Progress is specifically pronounced in sub-Saharan African countries, where AIDS-associated mortality and new infection have declined by 29% and 12%, respectively, since 2010 [2].

Many people who start HAART discontinue treatment, undermining the morbidity, mortality and prevention benefits of therapy. Stigma and discrimination, lack of psychosocial support, inaccessibility to services, opportunistic infections, and drug side effects are all commonly-sited reasons for discontinuing therapy [3]. Attrition varies at different times since initiation of therapy. A multi-site assessment conducted in low- and middle- income countries estimated average retention at 12, 24, and 60 months post-initiation was 81%, 75%, and 67%, respectively [4]. Attrition in HAART in Ethiopia appears to vary similarly, but data are limited. In four health centers in Tigray region, retention was 92% and 85% at 6 and 12 months, respectively. Variation was also present between facilities; in the sites evaluated, 12-month retention ranged from 78 to 92% [5].

Many clients restart treatment on their own after an initial episode of discontinuation. But for those who do not reinitiate treatment independently, supportive services including phone-based patient tracking and home visits from peer supporters or health care workers may be conducted to encourage re-engagement. Even with supportive mechanisms such as patient tracking, not all clients re-engage in care, and those who re-engage may subsequently exit treatment again. Treatment interruption practices among those who reinitiate therapy after discontinuation were described in a study in Uganda, where 43% of re-starters were lost to follow-up (LTFU) within 18 months of reinitiating treatment [6]. Although it’s possible similar trends may be present in chronic HIV care settings in Ethiopia, treatment discontinuation and attrition patterns have not been described in this setting. The objective of this study is to describe treatment interruption among HAART re-starters, to examine longer-term trends in engagement and loss from care among re-starters, as well as to determine clinical factors associated with treatment interruption.

## Methods

### Study setting

This study is conducted in ten randomly selected hospitals from among 38 hospitals in Ethiopia located in Addis Ababa, Benishangul Gumuz, Gambella, and Southern Nations Nationalities and Peoples Region (SNNP). The cumulative total of unique patients who had ever started HAART in the selected facilities combined was over 22,700 in 2014. Fee-based HAART was available before 2005, when free HAART services became available nationwide [7]. Follow-up services were customized to clients according to their need, ranging from every month to every 3 months. Follow-up was made by doctors, health officers or nurses trained in management of chronic HIV care, treatment and support. Starting in 2007, peer educators or “adherence supporters” were available to assist medical providers with providing adherence counseling to patients and conducting patient tracking following missed appointments or possible loss to follow-up [8]. Clinical data was updated in registers, medical records, and electronic databases, kept in secure and confidential locations. The most common antiretroviral medications used were as follows: First-line HAART was a combination of stavudine, zidovudine, abacavir or tenofovir plus lamivudine plus neverapine or efavirenz; Second-line regimen contained lopinavir/ritonavir in the place of neverapine or efavirenz [9]. A nationally-standardized system for monitoring and evaluation was in place at every study site. At the time of treatment, clinicians documented care and therapy in personal medical records. Relevant data from medical records were then transferred to facility registers and electronic database by data clerks. Two data clerks trained specifically in the management of HIV care and treatment data were maintained at antiretroviral (ART) treatment clinics to track and update records and documentation, to alleviate work load among clinicians. Data quality assurance measures, supervision and mentorship programs for clinicians and data clerks were in place to ensure data was properly generated, recorded and used onsite. Information with regard to patient tracking was recorded by peer educators, and was then reconciled with individual medical records, facility registers and electronic database at ART clinics. Pharmacy records at facilities were also consulted to cross-check medication pick-up data from medical records.

### Study design

This is an observational retrospective cohort study, using medical records documented as part of the routine care from clients who started HAART in selected facilities in Ethiopia from September 2005 to November 2013. Data was captured electronically from the patient records. Clients who started treatment at other facilities but received follow-up at a study facility (transfers) were excluded. From the full cohort, we evaluated the average time to 1^st^ episode of treatment interruption, average time to restarting treatment after the 1^st^ episode of treatment interruption, and average time to 2^nd^ episode of treatment interruption. We identified clients who had a documented episode of treatment interruption followed by restarting treatment (“restarters”, and compared them with clients with un-interrupted treatment (“continuous treated”), to compare longer-term outcomes over specific comparable time periods in individual treatment history. For example, all clients who discontinued treatment before month six of HAART treatment but restarted treatment by month six or before, were compared with those who never discontinued treatment by month six of follow-up. Subsequent treatment interruptions were determined and compared among restarters and continuously treated, for a maximum duration of 60 months of follow-up. In order to examine patterns of treatment interruption for clients who restarted treatment at different time periods in treatment history, the same comparisons were made between restarters who re-engaged in care matched with continuously-treated individuals at months 12, 18 and 24. Finally, predictors of retention in treatment were examined to identify differentiating demographic and clinical characteristics among restarters to identify those who were more likely to remain in care.

### Operational definitions

**Treatment interruption** was defined as having terminated HAART treatment for more than 1 month. This could be due to being lost from care at treatment initiating health facility, decision to stop treatment, or death.

**Loss** was defined as failure to present for HAART medication refill at the treatment-initiating health facility, with inability to be traced back by phone or home visit for more than 1 month, without a documented reason for failing to present (eg. no confirmed death nor decision to stop treatment in agreement with health care worker) [10].

**Death** was defined as a known client death from any cause, confirmed by health care worker or post-loss tracking.

**Stop** was defined as discontinuation of HAART in agreement with health care worker at treatment-initiating health facility.

**Restart** was defined as resuming treatment at the treatment-initiating health facility after treatment interruption.

**Retention** was defined as active antiretroviral treatment therapy at the treatment- initiating health facility.

**Favorable treatment outcome** was defined as being active in treatment up to 60 months post-HAART initiation, or up until September 2014 (whichever came first), at treatment- initiating health facility.

**Unfavorable treatment outcome** was defined as being classified as lost, dead, or stopped from treatment following agreement with treating health care worker, at the last contact visit prior to 60 months post-HAART initiation at treatment-initiating health facility.

**Transfer out**: when a patient is referred from the facility where s/he started ART to another health facility.

**Transfer in**: when a patient is received from another health facility after s/he is started on ART at that facility.

### Variables and data collection

The following variables were abstracted from patient medical records and included in study: age at treatment initiation, gender, baseline WHO Stage, baseline CD4 cell count, HAART start date, date of each follow-up, HAART treatment status (active, lost, dead, stop, transfer out, transfer in). The primary outcomes of interest were unfavorable treatment outcome on or before month-60 of follow-up, from the date of re-initiation among re-starters and the comparable date for the comparison group of continuously-treated. Patient medical record data was ascertained from an MS Access electronic database kept at the health facilities selected as study sites, for the purpose of generating routine monthly reports. Data entry was made from paper based medical records daily by trained data clerks. Data quality assessment was made routinely onsite as well as by external mentors.

### Statistical analysis

Data cleaning and analyses were conducted using STATA version 11 (Stata Corp, College Station, TX, USA) statistical software. Time to 1^st^ episode of treatment interruption, time to restart, and time to 2^nd^ episode of treatment interruption were described using mean, median, and inter-quartile ranges, and were plotted graphically using histograms. Survival analysis was carried out to compare the retention experience between re-starters and those that had not discontinued treatment. Clients were uncensored at the time of loss, death or stopped treatment (unfavorable treatment outcome). Those remaining active in treatment engagement at 60 months of follow-up or at September 2014 (whichever came first) were considered to have had favorable treatment outcome, and were censored at that time. Regression analysis was made using Cox proportional hazards model. Within-site correlation of patient characteristics were controlled by stratification. α was set to be 0.05 for all analyses [11, 12]. The dataset is available as Additional file 1.

## Results

### Baseline characteristics

The total study population included 22,647 unique individuals. The median follow-up time was 2.7 years (inter-quartile range (IQR): 8 months–6 years). Most study participants were adults (98%), and females accounted for 54%. The median age was 34 years (IQR: 28–40 years). Most clients, approximately 68%, started treatment in WHO clinical stage III or IV. Median baseline CD4 cell count was 148 (range: 65–195/mm^3^). Additional baseline characteristics are presented in Table 1 below.

**Table 1:** Baseline Characteristics (*n* = 22,647)

Variable	Sub-category	Number (%)
Age	<15	513 (2)
	≥15	22,134 (98)
Gender	Female	12,226 (54)
	Male	10,421 (46)
Baseline WHO Stage	I or II	7,286 (32)
	III or IV	15,361 (68)
Baseline CD4	<100	8,445 (37)
	100–349	12,384 (55)
	≥350	1,818 (8)
Ever discontinued ART	No	13,888 (61)
	Yes	8,759 (39)

### Treatment interruption

**Fig 1:**
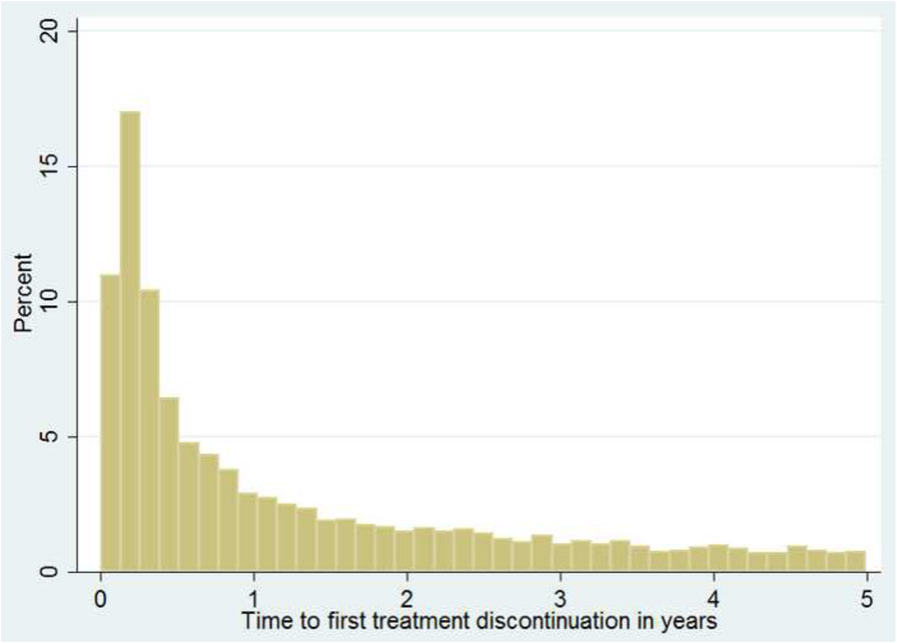
Time to 1^st^ treatment interruption (years)

**Fig 2:**
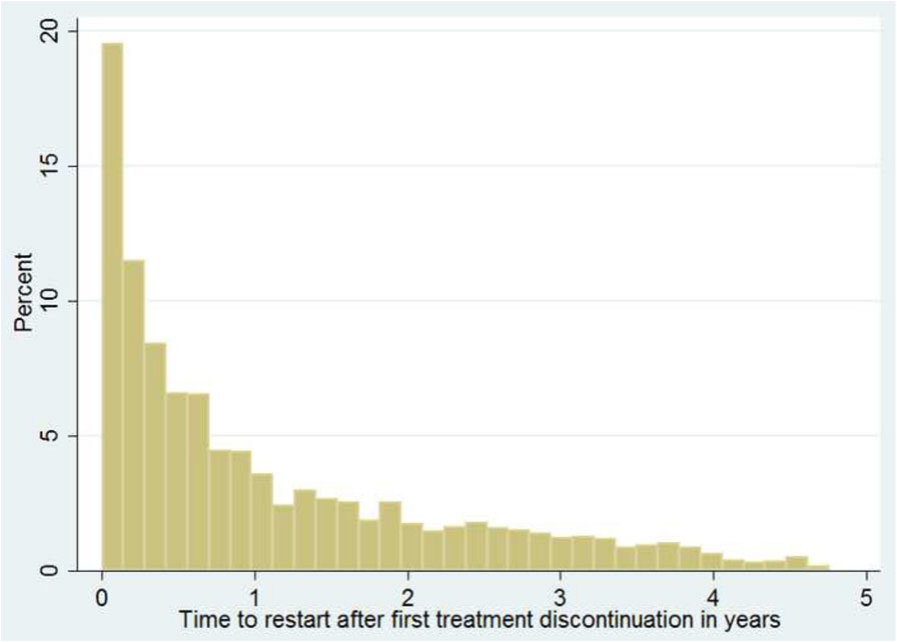
Time to treatment restart after 1^st^ loss (years)

**Fig 3:**
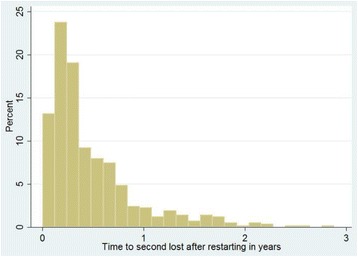
Time to 2^nd^ interruption after restart (years)

Approximately 39% of clients had discontinued treatment for a month or more at least once throughout the duration of follow up (8,759) (see Table 1). Among clients that had discontinued at least once, the median time to first discontinuance was one year (IQR: 0.3–2.6 years). Only 35% (*n* = 3,061) of clients restarted HAART after the first interruption following enrollment. Among those who did restart, median time to restarting treatment was 7 months (IQR: 2.2 months–1.7 years). Figure 1 depicts the distribution of time to 1^st^ treatment interruption. A substantial number of clients discontinued treatment in the first few weeks after starting HAART. Similar trends are seen for time to return to treatment (Fig. 2). Of those who restarted treatment, 24% (735) discontinued treatment for second time over follow-up. Half, or 50% of the cases who discontinued a second time, had done so within five months of follow-up (Fig. 3).

### Follow-up outcome

Unfavorable treatment status at the end of observation was observed for 28.5% (6,459) of the study population: 2,188 (34%) died, and 4,271 (66%) were lost to follow-up. Compared to individuals that were active in treatment at 6 months, those that had discontinued but restarted treatment within the first 6 months were 1.9 times (95% confidence interval 1.5–2.4) more likely to have an unfavorable outcome at the end of 5 years. One-year retention in care was 83% for those that had discontinued and re-started before six months, while it was 91% for those that had never discontinued. At the end of follow-up, retention was 57% for restarters within 6 months, and 76% among comparable individuals who had not discontinued by 6 months. Being adult, male, having higher WHO stage (III or IV) and having lower CD4 cell count at baseline were associated with higher hazard of unfavorable treatment outcome at the end of follow-up. Similar patterns were observed for assessments at months 12, 18 and 24. For all time periods assessed, re-starters experienced more than two-fold excess hazard of unfavorable outcome (HR = 2.4 (95% confidence interval 2.0–2.8), HR = 2.6 (95% confidence interval 2.2–3.1), and HR = 2.4 (95% confidence interval 2.0–2.8), respectively). (Tables 2 and 3) Among those who discontinued and restarted treatment, being male, being WHO stage (III or IV), discontinuing treatment for the first time before 6 months of follow-up, and restarting treatment within 6 months of interruption were associated with unfavorable treatment outcome at the end of follow-up (Table 4).

**Table 2:** Determinants of unfavorable treatment outcome at 60 months among those active in treatment at month six

Variable	Sub-category	Hazard ratio, crude	*P*- value	Hazard ratio, adj	*P*-value
Age	<15	1		1	
	≥15	1.6	0.001	1.6	0.002
Gender	Female	1		1	
	Male	1.3	<0.001	1.2	<0.001
Baseline WHO Stage	I or II	1		1	
	III or IV	1.5	<0.001	1.4	<0.001
Baseline CD4	<100	1		1	
	100–349	0.7	<0.001	0.8	<0.001
	≥350	0.8	0.008	0.9	0.106
Ever discontinued HAART before 6 months	No	1		1	
	Yes	1.8	0.000	1.9	0.000

**Table 3:** Hazard of unfavorable treatment outcome at the end of follow-up among those active in HAART care at months 6, 12, 18 and 24 months

Variable	Sub-category	Hazard ratio, adj	*P*-value
Ever discontinued HAART before 6 months	No	1	
	Yes	1.9	0.003
Ever discontinued HAART before 12 months	No	1	
	Yes	2.4	<0.001
Ever discontinued HAART before 18 months	No	1	
	Yes	2.6	<0.001
Ever discontinued ART before 24 months	No	1	
	Yes	2.4	<0.001

**Table 4:** Determinants of unfavorable treatment outcome at the end of follow-up among those who restarted treatment for HAART after treatment interruption (*n* = 3,922)

Variable	Sub-category	Hazard ratio, crude	*P*- value	Hazard ratio, adj	*P*-value
Age	<15	1			
	≥15	1.4	0.300		
Gender	Female	1		1	
	Male	1.6	<0.001	1.6	<0.001
Baseline WHO Stage	I or II	1		1	
	III or IV	1.2	0.061	1.3	0.014
Baseline CD4	<100	1			
	100–349	0.8	0.049	0.9	0.138
	≥350	0.8	0.112	0.8	0.178
Time to 1^st^ interruption	<6 months	1		1	
	≥ 6 months	0.4	<0.001	0.3	<0.001
Time to 1^st^ restart	< 6 months	1		1	
	≥ 6 months	0.3	<0.001	0.2	<0.001

## Discussion

Treatment interruptions were common in the study population. A substantial proportion (39%), of clients who started HAART in the study facilities discontinued treatment at least once. Of these, 35% restarted treatment, but approximately a quarter of those discontinued treatment again. Among those who discontinued and re-started, unfavorable outcomes at the end of follow-up were at least twice as likely as among those who had never discontinued treatment at months 6, 12, 18, and 24.

Estimates of retention in care, or active engagement on HAART among patients who have initiated therapy, vary in different settings. In an observational, prospective, multi-site cohort study of HIV-infected patients who initiated ART for the first time in Tigray, Ethiopia, the 12 month retention was 85%. In a different multi-clinic observational study in three regions in Ethiopia, at 3 years, survival among 93,418 patients on HAART was 70% [5, 13]. Estimates of survival from the current study are comparable. But, only a few studies have assessed long term treatment outcome among those who interrupt and restart therapy. A study in Uganda [6] found that of clients who interrupted but subsequently resumed treatment, only 52% were active in care after 18 months. This is similar with the current study’s estimate of 56%. In the Uganda study, those who returned to care on their own were more likely to resume care after second treatment interruption than were those who were traced by health care workers and re-engaged: 61% vs. 39%. In our study, analysis indicates that those that discontinued and restarted again earlier were more likely to have unfavorable outcomes in the long term. It is possible that re-starters that were temporarily lost from care may tend to have barriers to care that remain unaddressed upon re-entry, undermining subsequent adherence to treatment. These may be related to lack of access to care and support programs, distance from treatment site, advanced disease stage, or lack of proper adherence counseling [14, 15]. A study in British Columbia [16] describes a slightly different trend in treatment interruption experiences. In this setting, only 29% of patients were active on treatment continuously, though the follow-up period was longer than the current study (1996–2012). However, in contrast to our study, patients of younger age, higher CD4 count, and earlier WHO clinical stages were more likely to be lost. It may be that healthier patients may not feel the need to adhere to treatment, as their present health status may give them a false assurance of health. In our study, however, we found the reverse: sicker clients were more likely to discontinue follow-up. The explanation for this could be that sicker people may be unable to ambulate easily and struggle to travel to attend clinic and acquire medication [13].

This study clearly describes the distribution of time between treatment initiation and interruption, among treatment interrupters. Clients were generally more likely to interrupt medication in early stages of treatment than later stages. This may be due to drug side effects, resurgence of opportunistic infections, non-disclosure of status or fear of stigma and discrimination [17–20]. But, the time to the first occurrence of treatment interruption was 1 year, as compared to an average of 5 months to next occurrence after restarting. This reduction in time on treatment may be an indication of adherence fatigue [21]. Or as explained earlier, recurrence of problems that were unaddressed the first time treatment was interrupted. This points to the need for a more targeted intervention to help clients achieve long-term treatment goals, including viral suppression. In most high burden facilities where providers are burdened with a high work load, treatment supporters are available to assist with counseling of patients. Counseling may be targeted to support those with a pattern of poor adherence, by sharing personal experiences and teaching ways to cope with challenges [22–24]. Peer supporters may also be able to reduce the length of interruption before restarting treatment.

The median time taken to restart treatment in this study was seven months, although nearly a third of those who restarted did so in the first few weeks. Health care workers may be able to do more to reduce this time by preparing patients before re-starting treatment, and addressing emerging challenges as they appear. Improving accessibility of care is one important approach to reducing loss from care. Bringing treatment closer to patients’ vicinity may alleviate barriers, but such approaches may be complex to introduce, and may not result in improvements in engagement in all settings. In a study conducted by Médecins Sans Frontières in 25 HAART treatment programs in multiple countries in Africa and Asia, program scale-up to larger geographic coverage was associated with more lost to follow-up, though it did result in improved mortality. The reduction in mortality was comparable to the incremental increase in loss rate. Further work may need to be done to identify and re-engage clients that are lost, which is more likely to happen with increased frequency as more people are initiated on HAART. As healthier patients are initiated earlier, it may be more difficult to encourage adherence to treatment. For test-and-treat strategy to result in mortality reduction, these barriers will need to be addressed [1, 3, 25].

Among re-starters, male gender, being in advanced WHO stage III or IV at baseline, history of treatment interruption in the first 6 months after starting HAART, and restarting treatment within 6 months of interruption were significant predictors of subsequent loss from care. We believe other factors may be helpful in identifying those at risk of loss. This study used existing electronic medical records and data, and therefore was limited to the information that was previously collected. We were therefore unable to explore other potential parameters of interest. More studies are recommended, to inform the development of a proper tool to help prioritize clients at risk, so that health care workers can target those most in need of support.

Our analysis assumed that all lost patients interrupted treatment, when in fact there is a possibility that some restarted treatment at a different facility. This was demonstrated in one study where nearly 20% of patients considered lost were found to be on treatment at a different facility [26]. Therefore, our analysis represents the “worst case scenario” and may over-estimate loss from treatment. The strength of this study comes from the large number of clients followed over a long duration of time, including from the beginning of free HAART initiation in Ethiopia. This study relied on secondary data collected for program purposes. Although the original data may have had some errors or missing information, we took utmost precaution in our data abstraction to maintain accuracy as originally recorded.

## Conclusion

This study indicates that treatment outcomes of HAART defaulters are often unfavorable, and provides evidence of the need for approaches to improve retention in care. Those who interrupt treatment follow-up and then return to care experience less frequent re-engagement upon subsequent interruptions from care. Frequent delayed return to care and early relapse indicate that there is much room for improvement in patient preparation and tracing as treatment coverage expands further and healthier clients initiate treatment.

## Additional file

Additional file 1: Excel file containing study dataset used for analysis. (XLS 3617 kb)

## Abbreviations

CD4: Cluster differentiation 4
HAART: Highly active antiretroviral therapy
HIV: Human-immune deficiency
LTFU: Lost to follow-up
WHO: World health organization
